# *Cryptosporidium muris* Infection in an HIV-Infected Adult, Kenya

**DOI:** 10.3201/eid0802.010256

**Published:** 2002-02

**Authors:** Wangeci Gatei, Richard W. Ashford, Nicholas J. Beeching, S. Kang'ethe Kamwati, Julie Greensill, C. Anthony Hart

**Affiliations:** * Liverpool School of Tropical Medicine, Liverpool, United Kingdom; † University of Liverpool, Liverpool, United Kingdom; ‡ Kenya Medical Research Institute, Nairobi, Kenya

**Keywords:** Cryptosporidium, *C. muris*, human infection, genotypes

## Abstract

We describe a case of *Cryptosporidium muris* infection in an HIV-infected adult with diarrhea in Kenya. Sequence analysis of an 840-bp region of the 18S rRNA gene locus demonstrated the isolate had 100% nucleotide identity with *C. muris* recovered from a rock hyrax, 98.8% with a *C. muris* “calf” isolate, 95.5% with *C. serpentis*, but only 87.8% with *C. parvum* “human” type.

Tyzzer identified the first *Cryptosporidium* species, *C. muris*, in the gastric glands of mice (*1*). Thereafter, he identified *C. parvum*, which infects the small intestines of many mammals, and described the complete coccidian life cycle. Over the next 70 years, more than 23 different species of the genus were described on the basis of their morphology and natural hosts. However, when animals were experimentally infected, many of the described *Cryptosporidium* species were found to be identical. In the 1970s the classification was revised, and today only six to eight species are recognized as valid, with most human, zoonotic, and mammalian infections being attributed to the different *C. parvum* genotypes (*2*,*3*)*. C. muris*, which is naturally a murine parasite, appears to have a more limited host range than *C. parvum*. Experimental transmission studies of *C. muris* have shown that the isolate from laboratory mice can infect other animals, including dogs, guinea pigs, rabbits, lambs, and gerbils, although it did not produce patent infections (*4*). The parasite has also been isolated from a rock hyrax (*Procavia* sp.) from a zoo and a Bactrian camel with chronic cryptosporidiosis (*3*,*5*). For many years, the parasite was thought to infect cattle; however, recent studies have shown that the *C. muris* that infects cattle is genetically distinct, and a new species name, *C. andersoni*, has been suggested (*6*).

Conventional diagnostic methods for *Cryptosporidium* do not differentiate the various species and genotypes, and most infections are diagnosed as *C. parvum*. *C. parvum* “human” and “bovine” genotypes remain the main causes of human cryptosporidiosis, but lately identification of infections with other genotypes and also *Cryptosporidium* species other than *C. parvum* has increased in both immunosuppressed and immunocompetent persons (*7*–*10*). Possible asymptomatic human infection with *C. muris* was reported in two healthy girls in Indonesia (*11*). Morphologic studies on the oocysts showed they were most likely to be *C. muris,* although there was no genotypic or experimental animal confirmation. Phylogenetic analysis has enabled more conclusive assignment to species and genotypes infecting humans and other animals (*12*,*13*). We report a case of *C. muris* infection, confirmed by morphology and genotyping, in an adult HIV-infected man from Kenya, hospitalized with diarrhea.

## The Study

Fecal samples were collected from diarrheal patients from a hospital in Nairobi, Kenya, as part of a larger study. The patient described was an HIV-infected man who had clinical tuberculosis and diarrhea. *Isospora belli* was also detected in a fecal sample from the patient.

The fecal samples were preserved in both sodium acetate formalin and 2.5% potassium dichromate and kept at 4°C. They were stained with Kinyoun’s carbol fuchsin modified acid-fast stain and examined by oil immersion microscopy . An aliquot of 400 μL of the sample suspension in 2.5% potassium dichromate was processed for genotypic analysis. Potassium dichromate was washed 5 times with cold, distilled water until the yellow color cleared. Oocysts were ruptured by freeze-thaw, and DNA was extracted by using a QIAamp DNA Mini Kit (Qiagen, West Sussex, UK)) for stool DNA purification as per protocol.

A section of the SSU (18S) rRNA gene was amplified by nested polymerase chain reaction (PCR) as described (*14*), using the forward primers 5´-TTCTAGAGCTAATACATGCG-3´ and the reverse primer 5´-CCCTAATCCTTCGAAACAGGA-3´ for primary PCR. Secondary PCR used primers 5´-GGAAGGGTTGTATTTATTAGATAAAG-3´ and reverse primer 5´-AAGGAGTAAGGAACAACCTCCA-3´, employing the Techne (FTGENE2D Techne, Cambridge Ltd., UK) thermal cycler. Restriction fragment analysis of the secondary PCR product was done by digesting 15 μL of product in a 40-_L total reaction volume consisting of 15 U of *Ssp*1 and 3 μL of restriction buffer (Boehringer Mannheim Biochemicals, Indianapolis, IN) for species identification and *Asn*1 (Boehringer Mannheim) for genotyping in the same concentration at 37°C for 1 hour. Digestion products were separated on 2% agarose gel and visualized by ethidium bromide staining. The internal (secondary) fragment was purified by using the Prep-A-Gene DNA purification kit and cloned into PGEM-T Easy plasmid vector (Promega Corporation, Madison, WI) as described by the manufacturer. The cloned product was sequenced and aligned with previously published sequences of the 18S rRNA gene of *Cryptosporidium* species by using the CLUSTALX (EMBL, Heidelberg, Germany) program and manual adjustments. Multiple alignment was done with the Phylogeny Inference Package (PHYLIP version 3.5c, J. Felsenstein and the University of Washington, Seattle, WA). Sequences were analyzed by using DNADIST followed by neighbor joining (NEIGHBOR, PHYLIP package). One hundred replica samplings were analyzed for percentage bootstrap values. Accession numbers for *Cryptosporidium* 18S rRNA genes used were AF093498, AF093497, AF093496, AF108866 and AF093489, AF093499, AF112569 AF115377.

## Results

**Figure 1:**
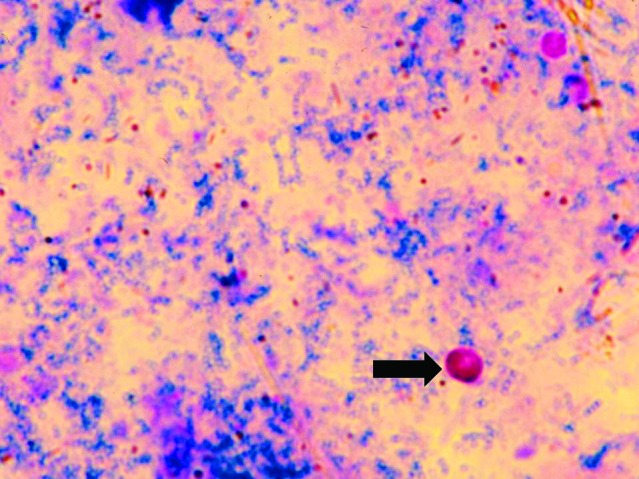
*Cryptosporidium muris* oocysts (under oil X 1,000), stained by Kinyoun’s acid-fast staining.

Microscopic examination of the acid-fast stained fecal smear revealed ovoid oocysts that were an average size of 7.5-9.8 x 5.5-7.0 μm (Figure 1). Cysts of *I. belli* were also identified in the stained smear.

Restriction endonuclease digestion with *Ssp*1 of the secondary PCR 18S rRNA product yielded two fragments of 385 bp and 448 bp in size, while *Asn*1 digestion yielded two visible bands that were 102 bp and 731 bp. The results match restriction fragment patterns observed following similar digestions of *C. muris* amplicons from a rock hyrax and Bactrian camel isolates (*14*).

**Figure 2:**
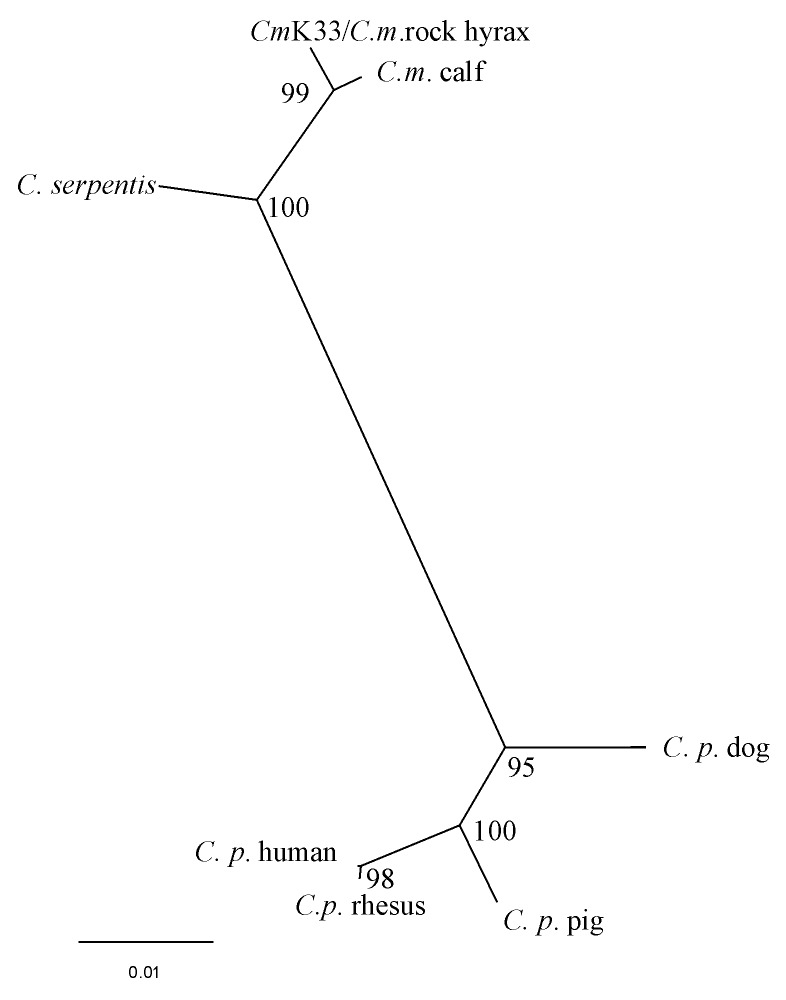
Comparison of 18S rRNA gene sequences of *Cryptosporidium* species. K33-*C.muris* from human patient (current paper Accession no. AJ307669); *C. m (muris*) “rock hyrax” (Accession no. AF093498); *C.m. (muris)* “calf”isolate (AF093496), now renamed *C. andersoni;* *C. serpentis* (AF093499); *C. p (parvum)* “dog” (AF112576); *C.p*. “rhesus monkey” (AF112569); *C.p.* “human” (AF093489); *C.p.* “pig” (AF115377). Numbers refer to the percentage of repeated analyses that gave the same tree topology (bootstrap values).

The resulting sequence 18S r RNA gene fragment of the *C. muris* human isolate was deposited in the EMBL Nucleotide Sequence Database (Accession no. AJ307669). Sequence analysis with ClustalX showed this human *C. muris* isolate had a 100% nucleotide identity to that of a *C. muris* isolate from a rock hyrax and a Bactrian camel (EMBL Accession no. AF093498, AF093497), 98.8% identity to a *C. muris “*calf” isolate (AF093496), 96.5% with *C. serpentis* (AF108866), and only 87.8% identity to *C. parvum* human type (AF093489). *C. muris* calf isolate (AF093496) has since been shown to be a different species from *C. muris* (“mouse” type, Accession no. AF093498 ) and has been given a new name, *C. andersoni*. The phylogenetic tree showed topology similar to that already published for *Cryptosporidium,* with *C. parvum* clustering in one clade, and our patient’s sample and published sequences of *C. muris* (rock hyrax isolate)*, C. andersoni* (calf isolate), and *C. serpentis* clustered in another group (Figure 2).

## Conclusions

Our study used genotypic analysis to confirm microscopic detection of *Cryptosporidium* oocysts in fecal samples and indicated that *C. muris* can indeed infect humans. Although immunosuppression has been observed to produce an increased susceptibility to cryptosporidiosis, the range of *Cryptosporidia* that can cause human cryptosporidiosis is still being elucidated (*8*,*13*,*15*). Lately, novel genotypes and *non-C. parvum* species such as *C. meleagridis* , *C. felis,* and *C. parvum* “dog” type have been identified not only in HIV-infected persons but also in HIV-uninfected patients (*7*,*9*,*10*). Genotypic analysis of *Cryptosporidium* organisms in fecal samples in the United Kingdom showed the occurrence of *C. meleagridis, C. felis,* and *C. parvum* “dog” type in immunocompetent and immunosuppressed persons (*10*,*16*). In another study in Peru, *C. felis, C. parvum* “dog” type, and *C. meleagridis* were identified in children not infected with HIV. In that study, *C. meleagridis* was as common as *C. parvum* “bovine” type; it appeared to be a stable part of the enteric pathogen mix causing cryptosporidiosis, perhaps only being identified with current definitive molecular methods (*9*).

*C. muris* infects the gastric glands of immunocompetent or immunocompromised (nude and SCID) mice (*17*); however, since our patient was co-infected with *I. belli*, the role of *C. muris* in our patient’s gastroenteritis and its possible site of infection in this patient are unclear.

A report of possible asymptomatic *C. muris* infection in healthy persons (*11*) and our finding of it in an immunosuppressed patient suggest that this may be yet another *Cryptosporidium* species with a zoonotic potential. The range of animal reservoir hosts in which *C. muris* has been identified or experimentally transmitted adds to the importance of *Cryptosporidium* species as a public health concern (*3*,*4*,*15*). The current genotypic analyses are making it possible to make more conclusive diagnoses and to speculate on possible sources of infection (*14*–*16*). These techniques will need to be applied more widely to identify and characterize isolates of *Cryptosporidium* for more definitive epidemiologic mapping.
